# Recent Advances in Mass Spectrometry for the Identification of Neuro-chemicals and their Metabolites in Biofluids

**DOI:** 10.2174/1570159X11311040007

**Published:** 2013-07

**Authors:** Suresh Kumar Kailasa, Hui-Fen Wu

**Affiliations:** a Department of Applied Chemistry, S. V. National Institute of Technology, Surat – 395007, India;; b Department of Chemistry, National Sun Yat-Sen University, Kaohsiung, 80424, Taiwan;; c School of Pharmacy, College of Pharmacy, Kaohsiung Medical University, 800, Kaohsiung, Taiwan;; d Center for Nanoscience and Nanotechnology, National Sun Yat-Sen University, Kaohsiung, 80424, Taiwan;; e Doctoral Degree Program in Marine Biotechnology, National Sun Yat-Sen University, Kaohsiung 80424, Taiwan

**Keywords:** Neurochemicals, LC-MS, GC-MS, CE-MS, MALDI-MS.

## Abstract

Recently, mass spectrometric related techniques have been widely applied for the identification and quantification of neurochemicals and their metabolites in biofluids. This article presents an overview of mass spectrometric techniques applied in the detection of neurological substances and their metabolites from biological samples. In addition, the advances of chromatographic methods (LC, GC and CE) coupled with mass spectrometric techniques for analysis of neurochemicals in pharmaceutical and biological samples are also discussed.

## INTRODUCTION

Neuroscience is the subject to study the chemical composition and processes of the nervous system, which includes the brain, the spinal cord and the nerves and the effects of chemicals on them. As with all the senses, our perception of outside world is processed by the peripheral and central nervous systems. The human brain consists nearly 20 billion cortical neurons [1]. Neuron is a basic component in the human nervous system with different shapes and forms. The function of a neuron is to receive signals from the other neurons and to transfer signals to the cell body through the intracellular signal transduction pathways. Generally, a neuron contains many dendrites which connect to an average of 7000 other neurons [1]. The axon allows projections over a long distance (e.g. from the legs to the spinal cord). The synapses signal is produced by electrochemical pathways, which can release neuro-transmitters. Therefore, human brain is the most important and complicated organ to control the whole body functions. Thus, neurological disorders can lead to brain injuries as well as neurodegenerative disease. Today’s neuroscience research is focused on developing sensitive and specific tools for the identification of molecular species in biological tissues. A variety of neurological drugs have been synthesized and applied to treat neurological disorders. 

Neurological activities of these drugs are widely prescribed in the treatment of neurological disorders. Some of these drugs have also been frequently detected in emergency toxicology screening, drug abuse, and forensic medical examinations. Few drugs (TCAs) inhibit the reuptake of norepinephrine (desipramine, nortriptyline, and protriptyline secondary amines) and serotonin (amitriptyline, imipramine, clomipramine, and doxepine tertiary amines) in the central nervous system [2-4]. In order to detect their concentration from human fluids, tandem mass spectrometry has been widely used for sensitive identificaiton and confirmation of neurodrugs [5]. Note that the detection of neurodrugs and their metabolites is a challenging task to most analytical chemists due to a variety of factors such as compositional complexity, limited sample amounts and endogenous inferences. Therefore, review papers [5-9] and books [10, 11] have reported the functions of neurological drugs on the nervous system and their identification by using various analytical instruments including chromatographic and mass spectrometric tools. Prior to introducing these mass spectrometric platforms, we must briefly frame the types of neurological drugs, their generic and trade names, molecular weights and formulas, since many chemicals have been introduced as neurological drugs to treat neurological disorders. Due to space limitation, we provide one typical drug for each classification. Table **1** summarizes the classification of neurological drugs for generic names, molecular weights, structures, and trade names. 

In the past several decades, mass spectrometric techniques have been applied as the primary and effective analytical tools for the identification of a wide variety of molecules in the biocomplex samples. This is mainly attributed to their features allowing rapid, sensitive and routine analysis of minimal amounts/volumes of target analytes (typically femtomoles to attomoles) in complex mixtures. To date, MS has been recognized as the most important technique for the characterization of various molecules in biofluids due to many advantages such as high speed, sensitivity, selectivity and accuracy [12]. It separates 

charged ions, based on their mass-to-charge ratios (*m/z*) in the gas phase, by applying an electric or magnetic field and measures their relative abundance. It also can accurately measure molecular weights and structural information for many chemical speiceis with high sensitivity. Although it has excellent capability to analyze a wide variety of drugs in biological samples, sufficient sensitivity and selectivity are obtained by implementing a separation tool/technique prior to mass spectrometric analysis. In this paper, we introduce the recent advances in mass spectrometric methods for the identification of neurological substances from the following techniques: (i) extraction methods coupled with MS; (ii) chromatographic techniques coupled with MS; (iii) direct mass spectrometric tools for the identification of neurological chemicals in biofluids. 

## DETERMINATION OF NEUROCHEMICALS BY LC-MS RELATED TECHNIQUES

MS can measure the molecular masses of molecules precisely by converting them into gas-phase charged ions by using various methods such as electric filed and heat. Thus, the development of new methods for ion generation, mass analyzers, and new tools for data processing has made it possible to analyze many chemical substances such as small organic compounds, biomolecules, polymers, metal complexes and whole living cells/tissues by MS tools. Intensive reports from books [10, 11, 13, 14] and review papers [15-18] have introduced the developments of various ionization techniques including EI, CI, APCI, APPI, ESI and MALDI for the analysis of various classes of molecules including neurochemicals.

TCAs are mainly used for the treatment of psychiatric disorders such as depression, mainly endogenous major depression. TCA can act as an effective drug to control the serotonin and norepinephrine concentration to normal levels in the nervous system. However, it can cause serious side effects such as unbalancing of heart rate and blood pleasure. They are also frequently detected in emergency toxicological screening, drug abuse testing, and forensic medical examinations. Therefore, TCAs analysis is very important. Lancas’s group applied SPME coupled with LC-MS for analysis of TCAs (DMI, IM, NOR, AMT, and CL (internal standard)) in plasma samples [19]. The authors used polydimethylsiloxane/divinylbenzene (60 μm) coated fibers for SPME of TCAs at stirring rate 1200 rpm for 30 min at pH 11.0. The liquid chromatographic separation was performed by using RP-C_18_ column (150 mm × 2.1 mm, 5 μm particles) with AA buffer (0.01 mM, pH 5.50):ACN (50:50, *v/v*) as the mobile phase. The LOD was ~0.1 ng/mL for all TCAs. Similarly, SDME coupled with LC-ESI-MS/MS was used to determine the trace amount of AM and MA in serum [20]. The target analytes were effectively separated by using C_18_ reversed-phase column with ACN–water as a mobile phase. The LODs were 0.3 μg/L and 0.04 μg/L for AM and MA, respectively. Titier *et al*. reported a LC-MS method for the determination of selective serotonin reuptake inhibitors (FLU, PXT, SRT, FLV, and CTP), serotonin noradrenaline reuptake inhibitors (milnacipram and VEN), a noradrenergic and specific serotoninergic antidepressant (MIR) and five of their active metabolites (NF, DM-CTP, DDMCTP, DMVEN, and DMMIR) in blood [21]. The conventional LLE technique was used for the extraction of these drugs from blood and they were separated by using XTerra reverse-phase C_18_ column with a gradient of ACN/AF buffer (4 mM, pH 3.2). The separated analytes were identified by ESI-MS with MRM mode. The limit of quantification (LOQ) is 5 ng/mL for all analytes (except for venlafaxine and desmethylvenlafaxine: 20 ng/mL). Intra- and inter- day precisions were lower than 11% and the recoveries were between 70 and 90% except for DMMIR, DMVEN, milnacipram, and DDMCTP, respectively. Very recently, del Mar Ramírez Fernández’s group developed a rapid and selective UPLC-ESI-MS/MS method for simultaneous quantification of 27 antidepressants and metabolites (AMT, CTP, CL, DMI, DMCTP, DCL, DMDS, DMD, DMFLU,DMVEN, DDMCTP, DOS, DOX, DLX, FLU, FLV, IM, MAT, MIA, MIR, MOC, NOR, PXT, RBX, SRT, TRZ and VEN) in plasma [22]. In this method, 1-chlorobutane was used as the solvent for the extraction of antidepressant drugs from plasma and were separated by using a BEH (Ethylene Bridged Hybrid) C_18_ analytical column with gradient elution and then detected by ESI-MS/MS. The LOQs and LODs were 2.5 - 10 ng/mL and 0.2 - 10 ng/mL for all analytes. Using this method, 59% - 86% (RSD < 16%) recoveries of analytes were achieved in the plasma samples. Importantly, this method was successfully applied to analyze antidepressant drugs and their metabolites in clinical and forensic samples. Moreover, a rapid and sensitive HPLC coupled with ESI-MS method was developed for simultaneous determination of AMT and NOR in rat plasma [23]. In this method, samples were alkalified with NaOH (0.5 mM) and both drugs were extracted by using LLE with methyl *t*-butyl ether. This method was successfully applied to study the pharmacokinetics in rats after intravenous injection of amitriptyline hydrochloride. Huande’s group developed a method using SPE coupled with HPLC-ESI-MS for rapid and sensitive determination of four nontricyclic antidepressants (FLU, CTP, PXT and VEN) in human plasma [24]. This method has shown good linearity (5.0-1000.0 ng/mL) for all compounds with *R^2^* = 0.9900. 

LC–ESI-MS has commonly been used for the analysis of neurological drugs and neurotransmitters in biofluids. For example, Li’s group developed a sensitive HPLC-ESI-MS method for simultaneous determination of VEN and its three metabolites (ODV, NDV and DDV) from human plasma [25]. The analytes were extracted by using LLE along with estazolam as the internal standard. The effective HPLC separation was achieved by using water (AA, 30 mM, formic acid 2.6 mM and TFA 0.13 mM) and ACN (60:40, v/v) as solvents with a C_18_ column (250 mm x 4.6 mm, 5 microm, Thermo, Bds, Hypersil, USA). The analytes were eluted within 6 min and then detected by ESI-MS in the SIR mode. The calibration curves were linear in the ranges of 4.0-700 ng/ml, 2.0-900 ng/mL, 3.0-800 ng/mL and 2.0-700 ng/mL for VEN, ODV, NDV and DDV with *R^2 ^*> 0.9991, average extraction recoveries > 77% and the LODs were 0.4, 0.2, 0.3, and 0.2 ng/mL, respectively. The same group applied HPLC-ESI-MS for simultaneous (stereoselective) analysis of VEN and ODV enantiomers in human plasma using vancomycin chiral columns [26]. This method showed good linearity in the range of 5.0-400 ng/mL for S-(+)-VEN and R-(-)-VEN, 4.0-280 ng/mL for S-(+)-ODV and R-(-)-ODV with *R^2^* >0.999. Furthermore, Qin’s team developed a rapid, selective and sensitive UPLC-ESI-MS/MS method for simultaneous determination of VEN and ODV in human plasma [27]. Sample pretreatment was performed by using diethyl ether and the analytes were separated by using a C_18_ column (Acquity UPLC BEH) with AA (10 mM) and MeOH as the mobile phase. The separated analytes were detected by using a triple-quadrupole tandem mass spectrometer with MRM mode *via* the ESI ionization/interface. Moreover, HPLC-ESI-MS/MS methods have been developed for the simultaneous determinations of ENP and ENPT in human plasma using LLE [28-29] and 96-well SPE [30]. These methods showed LOQs in the range of 0.1 – 1.0 ng/mL for both ENP and ENPT. The intra- and inter-day precisions of these methods were 7.7 - 13.3 and 7.8 - 15.4% (%RSD) for ENP and ENPT, respectively. Recently, Ghosh’s group developed a rapid and sensitive method *via *SPE coupled with LC-ESI-MS/MS for simultaneous determination of ENP and its metabolite (ENPT) in human plasma [31]. The extracted analytes were separated by using a C_18_ column (50 mm × 4.6 mm, 5 µm) with an isocratic mobile phase and then detected by using ESI-MS/MS in the positive ion and MRM mode. The ENP and ENPT mass peaks appeared at *m/z* 377.10 → 234.20 and 349.20 → 206.10 and the calibration curves showed excellent linearity within the range of 0.064 - 431.806 ng/mL for ENP and 0.064 - 431.720 ng/mL for ENPT (*R^2^* ≥ 0.990), respectively. 

Monitoring acetylcholine in the brain regions is very important to understand the disease pathology and to design and evaluate possible disease-modifying treatments. It has been suggested that ACh plays a significant role in the modulation of tissue inflammation. Zhang’s group developed a sensitive and quantitative LC-ESI-MS/MS method for the analysis of ACh, Ch and iso-ACh in brain microdialysis samples of freely moving animals [32]. This method was successfully used to monitor ACh levels in its free form without having the use of cholinesterase inhibitor in the perfusate. Ion (cation) exchange chromatography was used to separate ACh, Ch, iso-ACh and related endogenous compounds with volatile elution of buffer that consisted AF, AA and ACN. The LODs were 0.2, 2.0 and 0.6 fM for ACh, Ch and iso-ACh, respectively. 

Microdialysis-based LC-ESI-MS is a powerful technique for *in vivo* detection of neurodrugs,neurological substances and neurotransmitters from brains. For example, Carrozzo *et al*., developed a LC-ESI-MS/MS method for quantitative analysis of acetylcholine in rat brain dialysates [33]. In this method, cation exchange chromatography was used for the separation of ACh, Ch, acetyl-β-methylcholine (IS) from endogenous compounds. The LODs were 0.05 and 3.75 fM for ACh and Ch, respectively. This method was successfully applied to evaluate the effect of oral administration of IDRA21, a positive modulators of AMPA receptor, on the release of ACh in the rat prefrontal cortex. Fu’s team described a HILIC coupled with ESI-MS/MS method for the separation and quantification of ACh in microdialysis samples of normal rats and of rats with local inflammation [34]. The mass transitions: *m/z* 146 → 87 for ACh and *m/z* 155→87 for the internal standard ACh-D9 were confirmed by low-energy ESI-MS/MS in the positive ion mode with MRM, Keski-Rahkonen and co-workers developed a rapid, simple and sensitive LC-APCI-MS/MS method for determination of ACh in microdialysis samples of rat brains [35]. The ACh was separated by using reversed-phase column with of isocratic conditions (2% (*v/v*) of ACN and 0.05% (*v/v*) of TFA) and then identified by a linear ion trap mass spectrometer with APCI source using SRM mode. Kennedy’s group published several papers on microdialysis coupled with capillary LC-ESI-MS/MS for determining enkephalins in the striatum of anesthetized and in freely-moving rats [36], of the endogenous ACh from the rodent brain *in vivo* [37] and of endogenous opioid peptides *in vivo* in the rat striatum [38], respectively. These methods were successfully used to measure the concentration of ACh and to determine neuropeptides such as met-enkephalin, leu-enkephalin, dynorphin A(1-8), and β-endorphin *in vivo*. The LODs were 1-2 pM, 0.04 nM and 0.5-60 pM for enkephalins, ACh and neuropeptides, respectively. 

Neurotransmitters (DA, 5-HT and NE) were successfully determined by LC-ESI-MS/MS [39]. This method was effectively utilized for the simultaneous measurement of neurotransmitters (DA, 5-HT and NE) and cocaine in brain dialysatest samples. The LODs were 200, 1000, 900 pM and 1 pg/mL for DA, NE, 5-HT and cocaine, respectively. Buck’s group described a rapid and reliable LC-ESI-MS/MS method for the determination of GABA and glutamate in brain microdialysates [40]. The analytes were separated by using a HILIC column with a binary gradient elution profile comprising of 0.1% formic acid in water and ACN. The analytes such as GABA, Glu as well as the respective internal standards [D(6)]-GABA and [D(5)]-glutamate were detected by ESI-MS/MS within 3 min. This method was further successfully applied to monitor the changes of the extracellular concentrations of GABA and Glu *in vivo* microdialysis in rats. Uutela *et al*. developed a sensitive LC-ESI-MS/MS method for the analysis of ACh and Ch in microdialysis samples in rat and mouse brains [41] and in moving rats [42]. A Ringer's solution (150 mM) was used to extract ACh and Ch from rat or mouse brain. In this method, ACh, Ch, and acetyl-β-methylcholine (internal standard), endogenous compounds and inorganic cations were separated based on their hydrophilic interaction with diol column and were eluted by using AF (20 mM, pH 3.3) and ACN (20:80, *v/v*). The eluted analytes were detected by ESI-MS/MS. This method was effectively applied to determine trace level of ACh (1.4fM) in rat brains . 

It was noticed that plasma free metanephrines were found to be the ideal biomarkers for the diagnosis of pheochromocytoma. Peaston’s group developed and validated the LC–ESI-MS/MS method for determination of plasma metanephrines (NMN, MN and 3-MT) and compared the diagnostic efficacy of the method with an enzyme immunoassay procedure in 151 patients [43]. It was found that 38 patients have pheochromocytoma. In this method, metanephrines were extracted and separated by using off-line SPE (96-well plate format) coupled with hydrophilic interaction chromatography and then identified by ESI-MS/MS. Similarly, Kozak’s team also described the applications of SPE technique coupled with LC-ESI-MS/MS for the monitoring of MN and NMN in plasma [44]. SPE was performed using C_18_ as the stationary phase with ion-pairing reagent and a porous graphitic carbon column and HILIC column was used for their separation with good resolution and with no interference from plasma matrix. The target analytes were identified by ESI-MS/MS. This method showed good linearity in the range of 7.2–486.8 and 18.0–989.1 pg/mL for MN and NMN, respectively. Clark and Frank illustrated the development, validation and implementation of a reliable high-throughput LC-ESI-MS/MS for identification of MN and NMN in urine [45]. The extracted analytes were separated by using a Restek perfluorophenyl column with formic acid (0.2%) in MeOH (5%) as a gradient cleanout step solution and with 50% of MeOH as a mobile phase. The analytes were directly detected by using a triple stage quadrupole (TSQ) MS (API 3200) with ESI in the positive mode and the LOD and LOQ were 2.5 and 10 nM for MN an NMN, respectively. 

Serotonin is naturally produced in the pineal gland which lies deep in the centre of the human brain. Generally, the adult human possesses 5 to 10 mg of serotonin in the intestine (90%) and the rest in blood platelets and the brain. It is a one of the 'wonder drug' and acts as a neurotransmitter. It plays numerous functions in the human body including the control of appetite, sleep, memory and learning, temperature regulation, mood, behavior, cardiovascular function, muscle contraction, endocrine regulation and depression. Guillén-Casla and coworkers developed a cLC–MS method for the analysis of serotonin (5-HT) and its precursors (5-HTP and TP) in chocolate samples [46]. The authors used acidic digestion for the extraction of target species in chocolate samples. The optimal cLC separation condition was achieved by using a mixture of ACN and AF (5 mM) (3:97, *v/v*; pH 4) as the mobile phase. The mass peaks were observed at *m*/*z* 177, 205 and 221 corresponding to 5-HT, TP and 5-HTP, respectively and the LODs were 0.01 - 0.11 μg/g for all analytes. These results revealed that serotonin and its precursors were found in 5 kinds of commonly consumed chocolates with different cocoa contents (70–100%) and the highest serotonin content was found in chocolate with a cocoa content of 85% (2.93 μg/g). Moreover, TP (13.27–13.34 μg/g) was found in chocolate samples with the lowest cocoa content (70–85%). Interestingly, 5-HTP was not identified in any chocolate samples. Huang and Mazza’s group described an analytical method for the simultaneous quantification of serotonin, MEL, *trans*- and *cis*-piceid, and *trans*- and *cis*-resveratrol by using LC–ESI-MS in both positive and negative ion modes [47]. The optimal analytical separation was achieved by using a mixture of ACN and water with formic acid (0.1%) as the mobile phase and then identified by ESI MS. This method was successfully applied to determine the serotonin, MEL, *trans*- and *cis*-piceid, and *trans*- and *cis*-resveratrol in 24 kinds of commonly consumed fruits. The highest serotonin content was found in plantain, while orange bell peppers had the highest melatonin content. It was noticed that grape samples contain higher *trans*- and *cis*-piceid, and *trans*- and *cis*-resveratrol contents than the other fruits. 

It has been confirmed that 5-HT in human platelet depleted plasma is used as a biomarker for the identification of functional gastrointestinal disorders. It acts as a neurotransmitter in the central and peripheral nervous systems in the body. Due to its key role, Monaghan’s team developed a simple and rapid LC–ESI-MS/MS for the quantification of 5-HT in plasma samples [48]. The 5-HT was extracted by using protein precipitation method and the solution was injected directly into a SecurityGuard SCX cation exchange column followed by isocratic elution into an Onyx Monolithic C_18_ analytical column. MeOH was used as the solvent for effective separation of analytes. The eluant was directly connected to a Quattro Premier XE ESI-MS/MS. The MRM transitions of analyte ions were observed at *m/z* 160→114.9 for 5-HT and at *m/z* 164.1→118.9 for d4-5-HT, respectively. This method was free from the interference (TP or 5-HIAA) and the LODs and LOQs were 1.5 and 5 nM, respectively. Very recently, Ansermot’ group described a simple and sensitive SPE coupled with LC-ESI-MS/MS method for simultaneous quantification of all selective serotonin reuptake inhibitors (CTP, FLU, FLV, PXT and SRT) and their active metabolites (DM-CTP and NF) in human plasma [49]. The stable isotope-labeled internal standard was used for each analyte to compensate for the global method variability and the analytes were extracted by SPE with mixed mode of Oasis MCX 96-well plate. The extracted analytes were separated within 9.0 min by using a XBridge C_18_ column (2.1 × 100 mm; 3.5 μm) with a gradient of AA (50 mM; pH 8.1) and ACN as the mobile phase. The separated analytes were identified by ESI-MS/MS. The method was successfully used to monitor routine therapeutic drugs in more than 1600 patients’ plasma samples over 9 months. This method was also suitable for both therapeutic drug monitoring as well as pharmacokinetic studies in the clinical laboratories. At the same time, Frenich and co-workers described a simple and sensitive UPLC-ESI-MS/MS method for the simultaneous determination of glutamate, GABA, Ch, ACh, DA, 5-HIAA, serotonin, DOPAC and HVA in rat brain [50]. The separation efficiency was greatly improved by adding HFBA into the mobile phase. The analytes were separated by a single chromatographic run (8 min) and then the analytes were identified by ESI-MS/MS in positive mode with MRM. This method showed good linearity with *R^2^* > 0.98 and the intra- and inter-day precision of the method (expressed as relative standard deviation) was < 26%. This method was successfully used to quantify the neurotransmitters in several rat brain regions (prefrontal cortex, striatum, nucleus accumbens and amygdala) and detected glutamate (1000 μg/g), GABA (30 μg/g) and Ch (100 μg/g) species in rat brain. 

Furey’s group described a versatile and validated method for the analysis of glutamate, GABA and Ch in urine using nano-ESI-MS^n^ interfaced with an LTQ Orbitrap mass spectrometer [51]. This method was successfully applied to analyze the target species without chromatographic separation. This method showed good linearity with *R^2^* = 0.9999 for serotonin and DA and 0.9955 for 5-HIAA, respectively. The LODs and LOQs were 9–12.9 nM and 27.2–57.7 nM for all analytes in urine. This method showed good intraday repeatability for all analytes with RSD values (n = 5) 4.4% - 6.2% and 2.1–8.1%, respectively. Precursor ions were confirmed by multiple tandem MS (MS^n^) with varying the energy of helium collision processes. The analytes were quantified by the identification of most intense ion transition for each compound and these were observed at *m/z* 177/160 (20%), 154/137 (23%) and 192/146 (35%) for serotonin, DA and 5-HIAA, respectively. The high energy CID scan event provides the more identification points for the analytes, as even more product ions are produced. In serotonin spectrum, the required optimum collision energy was 75% and yielded product ions at *m/z* 160, 132 and 115. For DA and 5-HIAA, the fragmented ions were observed at *m/z* 137, 119 and 91 for DA at 70% and at *m/z* 146, 173, and 118 for 5-HIAA at 90%, respectively. Kostiainen *et al*. developed LC-ESI-MS/MS method for the determination of intact GLUs, sulfates of common neurotransmitters serotonin, DA, 5-HIAA, DOPAC, and HVA in rat brain microdialysates [52]. The target analytes (5-HT-, 5-HIAA-, DOPAC-, and HVA-GLUs) were produced by enzyme-assisted synthesis method using rat liver microsomes as a biocatalyst. The other targets (sulfate conjugates) were synthesized chemically or enzymatically using a rat liver S9 fraction. In this study, for the first time, 5-HT-GLU was detected in rat brain. The results revealed that the concentration of 5-HT-glucuronide (1.0−1.7 nM) was 2.5 times higher than that of free 5-HT (0.4−2.1 nM) in rat brain microdialysates, whereas DA-GLU (1.0−1.4 nM) level was at the same or lower than the free DA (1.2−2.4 nM). Interestingly, the acidic metabolites of neurotransmitters (5-HIAA, HVA, and DOPAC) were found in free and sulfated form in rat brain microdialysates. The same group described the applications of LC−ESI-MS/MS for the quantification of dopamine and its phase I and phase II metabolites in brain microdialysis samples [53]. This method involves an enzymatic synthesis of target species using rat liver microsomes as biocatalysts where as dopamine glucuronide was used as a reference compound for their characterization. The authors confirmed the presence of dopamine glucuronide in rat and mouse brain microdialysis samples, which offers a detection limit of 0.8 nM. This method was successfully used to estimate the concentrations of DA and its glucuronide (2 nM) in the microdialysates of the striatum of rats brain. 

Neuroleptic (antipsychotic) drugs are tranquilizing psychiatric medication and are used to manage psychosis (including delusions or hallucinations, as well as disordered thought), particularly in schizophrenia and bipolar disorder. Weinmann’s group developed LC-ESI-MS/MS for the analysis of neuroleptics clozapine, flupentixol, haloperidol, penfluridol, thioridazine, and zuclopenthixol in hair samples of psychiatric patients [54]. The target drugs were extracted by ultrasonication with methanol, cleanup by SPE from hair samples and then identified by LC-MS/MS with MRM mode. This method was successfully applied to analyze the neuroleptic drugs in hair samples of psychiatric patients. Josefsson and co-workers reported a LC-ESI-MS/MS method for the determination of 19 most commonly prescribed neuroleptics in human tissues and body fluids such as blood, urine and hair [55]. This MS/MS method provided best platform for sensitive analysis of neuroleptics (LOD < 0.05ng/mL) in human tissues. 

Opiates are psychoactive chemical substances which bind to opioid receptors and play a key role in the central and peripheral nervous system and the gastrointestinal tract. Moreda-Piñeiro’s group developed a rapid and sensitive ESI-MS/MS method for simultaneous determination of MP, MAM, COD, COC and BZE in the hair of drug abusers [56]. This method involves an optimized matrix solid phase dispersion procedure with alumina, followed by dilute hydrochloric acid elution on SPE column for the clean-up/preconcentration of drugs from hair samples. Alternatively, ultrasound assisted enzymatic hydrolysis was performed with *Pronase E*, followed by an off-line SPE for clean up/preconcentration of target species. The extracted analytes were subjected to ESI-MS/MS with MRM for the identification and quantification of analytes. The method showed the highest sensitivity by delivering the targets with an ACN/water/formic acid (80/19.87/0.13, *v/v/v*) mixture. The LODs were 39.2, 4.4, 6.8, 7.0 and 7.4 ng/g for MP, MAM, COD, COC and BZE, respectively. Huestis *et al*. developed a LC-MS method for identification and quantification of MD, EDDP, COC, BZE, AMP, MP and COD in human umbilical cords [57]. SPE was used for the extraction of analytes from homogenized tissue (1 g). This method showed good linearity in the range of 2.5–500 ng/g for all analytes, except for MD (10–2000 ng/g). The method was effectively applied to analyze drugs in umbilical cords. It was found that illicit drug contains in ng/g, 40.3 (MP), 3.6 (COD), 442 (BZE), 186 (MD) and 45.9 (EDDP), respectively. Very recently, Saussereau’s group described a novel and facile approach for the quantitative determination of illicit drugs in dried blood spots with filter paper by LC-ESI-MS/MS [58]. Various opiates were used as illicit drugs (MP and its 3- and 6-GLU metabolites, COD, AMP), cocainics (ecgonine methylester, BZE, COC, CCE) and amphetamines (AM, MA). In this method, 30 μL of whole blood was spotted on A Whatman card 903 and dried at room temperature. From this, a 3-mm diameter disk was removed, suspended and then ultrasonicated with 150 μL of water for 10 min. The extracted analytes (100 μL) were identified by on-line LC–MS/MS. The optimal extraction and separation were achieved on Oasis HLB extraction column and C_18_ Atlantis analytical column with AF buffer (20 mM, pH 2.8) (solvent A) and ACN/solvent A (90:10, *v/v*) as the mobile phase. The recoveries of all analytes were up to 80% and this method was more suitable for the analysis of illicit drugs in whole blood with high sensitivity. 

LSD, iso-LSD, 2-oxo-3-hydroxy-LSD are potent psychoactive and hallucinogenic drugs for treatment on the central nervous system. In recent years, the use of LSD related chemicals is increasing worldwide, and the detection of LSD substances and its metabolites in body fluids continues to be a challenge because the small dosage (µg/kg) is used [59]. Therefore, the determination of various classes of LSD related drugs is important in many fields of analytical toxicology, such as forensic science, workspace drug testing, and antidoping analysis. Johansen and Jensen described a LC-ESI-MS/MS method for the determination of LSD, iso-LSD and 2-oxo-3-hydroxy-LSD in the forensic samples [60]. This method involves LLE for the extraction of analytes and LSD-D3 (internal standard) from 1.0 g of whole blood or 1.0 ml of urine using butyl acetate (pH 9.8). The extracted analytes were separated by LC and then detected by ESI-MS/MS (MRM mode). This method showed good linearity (0.01–50 μg/kg) and LOD (0.01μg/kg) for all transitions of LSD and iso-LSD. This method was successfully used to investigate the concentrations of LSD and iso-LSD (0.27 and 0.44 μg/kg) in the blood for a 26-year-old male suspected for having attempted homicide. Additionally, 2-oxo-3-hydroxy-LSD was detected in the urine and confirmed the presence of LSD in the blood of suspected person. Cailleux’s team described a LC-ESI-MS/MS method for quantification of LSD and iso-LSD by using one step LLE from blood and urine [61]. This method was applied to investigate the trace amounts of LSD and iso-LSD in two positive cases and detected the targets as follows, case 1: LSD=0.31 μg/L, iso-LSD=0.27 μg/L in plasma and LSD=1.30 μg/L, iso-LSD=0.82 μg/L in urine; case 2: LSD=0.24 μg/L, iso-LSD=0.6 μg/L in urine, respectively. This method was also employed for the quantification of the main metabolite (2-oxo-3-hydroxy-LSD) in urine and their concentrations were found to be 2.5 μg/L and 6.6 μg/L, respectively. Using this approach, the sensitivity of the method was greatly improved for the detection of Nor-LSD (0.15 and 0.01 μg/l) levels in urine and successfully identified Nor-iso-LSD, LAE, trioxylated-LSD, LEO and 13 and 14-hydroxy-LSD and their glucuronide conjugates in urine. 

Crompton’s group described an analytical procedure for the analysis of PCP in oral fluid by using LC-ESI-MS/MS [62], following initial screening with enzyme linked immunosorbent assay. This method applied SPE of PCP using the Quantisal ™ device and its quantification by APCI-MS. The LOQ was 5 ng/mL and the intra- and inter-day precisions were 3.04% and 3.35% (n=5) at concentration of 10 ng/mL and the percentage recovery of PCP was 81.7% (n = 6). Sergi and co-workers developed a micro-solid phase extraction coupled with LC-ESI-MS/MS technique for determining 11 drugs (PCP, AM, MA, MDOAM, MDOEAM, MDOMAM, COC, BZE, KT, MC, and PSC) in oral fluids [63]. In this method, target analytes were extracted by using MSPE with modified tips, made of a functionalized fiberglass with apolar chains of octadecylsilane into monolithic structure. The extracted analytes were identified by ESI-MS/MS. The LOQs were 0.3 ng/mL and 4.9 ng/mL for cocaine and psilocybine, with *R^2^* >0.99. Cannabis is the most commonly abused drug and is frequently quantified during urine drug testing. Very recently, Huestis’s group described the application of LC-ESI-MS/MS technique for the validation and quantification of THC, 11-OH-THC, THCCOOH, CBD, CBN, THC-GLU and THCCOOH-GLU in human urine (0.5 mL) [64]. The ideal separation was achieved by using ultra biphenyl column with a gradient of AA (10 mM, pH 6.15) and 15% MeOH in ACN at 0.4 mL/min flow rate. The analytes were identified by ESI-MS in both positive and negative ion modes. This method showed good linearity in the range of 0.5–50 ng/mL for THC-GLU, 1–100 ng/mL for THCCOOH, 11-OH-THC and CBD, 2–100 ng/mL for THC and CBN, and 5-500 ng/mL for THCCOOH-GLU with *R^2^* > 0.99. The average extraction efficiencies were 34–73% with analytical recovery (bias) 80.5–118.0% and showed with good total imprecision 3.0–10.2%. This method was effectively used for the simultaneous quantification of urinary cannabinoids and phase II glucuronide metabolites, and urinary cannabinoid glucuronides in various samples. 

Next, Dowling and Regan described a rapid and simple method for the analysis of CB substances (CP 47, 497) in urine by LC-ESI-MS/MS [65]. In this method, water-ACN (90:10, *v/v*) mixture was used for the separation of target analystes by LC. The LOD was 0.01 μg/mL and the RSD values were < 10%. Generally, the ionization efficiency of CBs is extremely low due to their non-polar nature, resulting in poor LODs. To solve this problem, Lacroix and Saussereau functionalized the phenolic groups of CBs with chloride dabsyl to form a product with a tertiary amine and then significantly improved the LODs of cannabinoids [66]. In this approach, LC-ESI-MS/MS technique was used for the quantitative determination of THC, 11-OH–THC, THC–COOH, CBN and CBD in microvolume blood samples. This method involves the protein precipitation followed by derivatization with dabsyl chloride and their analysis by LC-ESI-MS/MS. The optimum separation was achieved by using C_18_ analytical column (150 mm × 2.1 mm) with a gradient of water and ACN, which contained 0.2% of FA. The LOQs were 0.25, 0.30, 0.40 and 0.80 ng/mL for THC and THC–COOH, 11-OH–THC, CBN and CBD, respectively. 

Nicotine, caffeine and arecoline are the most consumed psychoactive drugs worldwide [67]. Among these, NIC is responsible for tobacco addiction, and it is the most specific component in the cigarette smokers. The biomarkers of NIC are suspected to contribute deseases such as cardiovascular and reproductive disorders in humans [68]. Large quantity of caffeine is consumed by pregnant and lactating mothers which is due to the prescription by the doctors and non-prescription drugs. ARC is the main alkaloid that is present up to 1% of dry weight. It acts as a stimulant to the central nervous system and shows psychoactive effects. Pichini’s group described the application of LC-ESI-MS/MS for the determination of NIC and its principal metabolites (COT, *trans*-OH-COT and CNO, CAF and ARC) in breast milk [69]. The target analytes were extracted by using LLE with chloroform/isopropanol (95:5, *v/v*) at neutral condition for NIC, COT, *trans*-OH-COT, CNO and CAF and at basic condition for ARC, respectively. The analytes were separated by using the C_8_ reversed-phase column with a gradient of AF (50mM, pH 5.0) and ACN as a mobile phase. Separated analytes were structurally identified by ESI-MS/MS. For NIC, the protonated NIC (at *m/z* 163) generated a major product ion at *m/z* 132 which is due to the loss of CH_3_NH_2_. Two other fragment ions were shown at *m/z* 120 and 106 produced by the losses of C_3_H: and C_3_H_7_N from *m/z* 163, respectively. For COT, the protonated ion ([M+H]^+^) shown at *m/z* 177 generated a major ion at *m/z* 80 due to the loss of the methylpyrrolidinone ring from COT. Other fragment pathways produced ions at *m/z* 98 and 146 corresponding to the loss of pyridine and CH_3_NH_2_ from COT. For ARC, the [M+H]^+^ ion at *m/z* 156 yielded the product ion at *m/z* 81 by the loss of CH_3_ and C_2_H_4_O_2_. Furthermore, molecular ion at *m/z* 156 yielded product ion at *m/z* 124 corresponding to the loss of CH_4_O and further loss of CO yielded fragmented ion at *m/z* 96. In addition, another fragment ion at *m/z* 141 was produced by the loss of CH_3_ form ARC. 

For CNO, the protonated ion ([M+H]^+^) was observed at *m/z* 193 and yielded the protonated pyridine *N*-oxide at *m/z* 96 by loss of the methylpyrrolidinone ring. In other fragment pathways, a product ion was produced at *m/z* 162 via the loss of CH_3_NH_2_, followed by the further loss of CO to give *m/z* 134 and the fragment ion at *m/z* 98 was generated by the loss of pyridine *N*-oxide. For *trans*-3-OH-COT, the protonated ion ([M+H]^+^) at *m/z* 193 generated a product ion at *m/z* 80 by the loss of the methylhydroxypyrrolidinone ring and the second characteristic ion at *m/z* 134 corresponding to the loss of C_2_H_3_O_2_ from the parent ion. The protonated OH-COT can also produce another product ion at *m/z* 162 by the loss of CH_3_NH_2_, followed by the further loss of CO_2_ to give *m/z* 118. For CAF, the major product ion at *m/z* 138 corresponding to the loss of C_2_H_3_NO from the protonated CAF at *m/z* 195 and further fragment ion at *m/z* 110 by the loss of CO from the product ion at *m/z* 138. The protonated molecule yielded product ions at *m/z* 180 (*via *the loss of CH_3_) and *m/z* 150 (via the loss of three CH_3_ groups). This method showed good linearity for all the analytes with *R^2^* > 0.998. This approach also provides excellent sensitivity for simultaneous quantification of NIC and its metabolites in breast milk. 

Recent years, several researchers have addressed the potential applications of liquid chromatography coupled with mass spectrometric techniques for the analysis of NIC, COT and its metabolites in various samples [70-83]. NIC, COT and *trans*-OH-COT are tobacco biomarkers in meconium. Xia’s group developed a SPE coupled with LC-ESI-MS/MS method for the analysis of *trans*-OH-COT, COT and NIC in meconium [70]. In this method, target species were analyzed with high precision 4.8–10.6%, 3.4–11.6% and 9.3–15.8% for *trans*-OH-COT, COT and NIC (intra- and inter-day) and with accuracy −4.0, 2.0 and 0.8% for *trans*-OH-COT, COT and NIC at 0.5, 2.5 and 7.5 ng/g, respectively. This method was successfully used to analyze NIC-related substance in 374 meconium samples from infants of both smoking and nonsmoking mothers. NIC, COT, *trans*-OH-COT and NC were effectively analyzed by LC-MS/MS in human plasma [71]. The average intra- and inter-assay analytical recoveries were between 101.9% and 116.8% for all analytes with the RSD values <11%. So far, there are no analytical methods for the simultaneously analysis of NIC, COT, *trans*-OHCOT, NN and NC in human meconium. Huestis’s team developed a LC-APCI-MS/MS method for simultaneous determinations of the above analytes in human meconium [72]. Homo-genization, enzyme hydrolysis and SPE methods were used to extract these analytes. This method was successfully used to evaluate the nicotine-related substances in human meconium which were collected from an tobacco exposed infant. Similarly, Meger and co-workers described a LC-APCI-MS/MS method for direct analysis of NIC, COT, *trans*-OH-COT and their corresponding glucuronide conjugates as well as CNO, NC, and NNO in the urine of smokers [73]. In this method, deuterium-labeled nicotine, cotinine, and *trans*-3′-hydroxycotinine were used as internal standards. The target analytes were functionalized with glucuronides by using chemical (cotinine-*N*-glucuronide) or enzymatic (nicotine-*N*-glucuronide and *trans*-3′-hydroxycotinine-*O*-glucuronide) procedures. This method allows rapid and simultaneous determination of nicotine and eight of its major metabolites in urine of smokers with good precision and accuracy. Miller’s team described a novel LC-ESI-MS/MS method for simultaneous determination of nicotine-N-β-d-glucuronide, CNO, trans-OH-COT, NC, trans-NICO, COT, NN, NIC, AT, AB and cotinine-*N*-β-d-glucuronide in human plasma and urine [74]. SPE method was used for the extraction of target analytes and then analyzed by LC–ESI-MS/MS (MRM mode). This method provided an effective platform for extraction of target species with 52–88% in plasma and 51–118% in urine. This method was successfully applied to determine the nicotine and related substances in nicotine-abstinent human participants and to investigate pharma-cokinetics of nicotine in plasma. 

Dobrinas *et al*. developed a method using SPE coupled with LC-ESI-MS/MS for sensitive and simultaneous quantification of NIC and its metabolites (COT and *trans*-OHCOT and VCL) in human plasma [75]. The best separation was achieved by using hydrophilic interaction liquid chromatography column (HILIC BEH 2.1 × 100 mm, 1.7 μm) with AF buffer (10 mM, pH 3) and ACN as the mobile phase. The separated analytes were identified by ESI-MS/MS. This method was successfully applied to analyze NIC, its metabolites and VCL in more than 400 clinical plasma samples with trueness (86.2–113.6%), repeatability (1.9–12.3%) and intermediate precision (4.4–15.9%). Marclay and Saugy developed a method using LLE coupled with LC–ESI-MS/MS for detection and quantification of NIC and its principal metabolites (COT, *trans*-OH-COT, NNO and CNO) in urine of ice hockey players [76]. The method showed good linearity in the concentration ranges of 10–10,000 ng/mL for NIC, COT, *trans*-OH-COT and 10–5000 ng/mL for NNO and CNO, with *R^2^* >0.95. This method was successfully allowed to measure the prevalence of nicotine exposure during the 2009 Ice Hockey World Championships, 72 samples were collected and analyzed after 3 months storage and found that every urine sample contains nicotine and/or metabolites. Moreover, sensitive LC-ESI-MS/MS methods were used to quantify NIC and its metabolites in plasma, urine, and saliva of smokers, and secondhand smoke exposure in non-smokers [77, 78]. Furthermore, mecamylamine is a nicotine antagonist under investigation in combination with nicotine replacement for smoking treatment. A sensitive LC-ESI-MS/MS method was validated for the quantification of NIC, COT, *trans*-3-OHCOT, NC and MECA in human urine [79]. A Synergi PolarRP column (a gradient of formic acid (0.1%) in ACN) was used for the separation of target analytes and the analytes were identified by ESI-MS in positive mode with MRM. This method showed good linearity in the range of 1–500 ng/mL for NIC and NC, 0.5–500 ng/mL for *trans*-OHCOT, 0.2–500 ng/mL for COT, and 0.1–100 ng/mL for MECA with *R^2^* >0.99, and mean extraction efficiencies were 55.1–109.1% for all the analytes. Vieira-Brock’s group developed a simple and sensitive LC–ESI-MS/MS method for simultaneous quantification of NIC, COT, NN, NC, NIC-GLU, COT-GLU, NNO, CNO, *trans*-OHCOT, AB and AT in rat brain tissue [80]. In this method, SPE was used for the extraction of target analytes and were separated by using Discovery® HS F5 HPLC column and then analyzed by ESI-MS/MS with MRM mode. The extraction recoveries were 64% to 115% for all analytes and the intra- and inter-assay imprecisions and accuracy were ≤12.9% and ≥86% for all analytes, respectively. This method was successfully used for the sensitive determination of NIC biomarkers in rat brain. Kataoka and co-workers described a method using on-line (in-tube) SPME coupled with LC-ESI-MS/MS for rapid and sensitive detection of NIC, COT, NN, AB, and AT in human urine and saliva [81]. A Synergi 4u POLAR-RP 80A column and AF buffer (5 mM)/MeOH (55/45, *v/v*) were used for efficient separation of target species and then identified by ESI-MS/MS. This method showed good precision within-run and between-day with RSD 4.7% and 11.3% (n = 5), respectively. Using this method, 83% recoveries of nicotine, cotinine and related compounds were obtained in urine and saliva samples with RSD <7.1%. Recently, Knabbe’s group developed a stable isotope dilution UPLC method coupled with ESI-MS/MS for determination of NIC, COT, the major oxidative and pharmacologically less active metabolite of NIC in human urine [82]. In this method, nicotine-d4 and cotinine-d4 were used as internal standards in sample preparation and the measurement of NIC and COT was performed within 1.5-min run-time. The LODs were 0.7 and 0.4 μg/L for NIC and COT, and LOQs were 1.7 and 1.1 μg/L for NIC and COT, respectively. Using this method, nicotine metabolites were effectively identified in human urine samples (n = 98) with high accuracy and precision. Recently, Knottenbelt’s illustrated the potential application of hydro-philic interaction chromatography combined with Fourier transform mass spectrometry (FT-MS) for determination of nicotine exposure in dogs [83]. Using this method, nicotine was found in the hair of dogs belonging to smokers and was found to be absent from the hair of dogs belonging to non-smokers. Moreover, NNO, COT, NC and NNNO were detected in the hair of dogs belonging to smokers. 

Monitoring the neurological substances *in vivo* is very important in neuroscience because it allows to monitor the correlation of neurotransmission with behavior, disease state, and drug concentrations in the intact brain. Very recently, Kennedy’s group developed a HPLC–tandem mass spectrometric method that utilizes benzoyl chloride for determination of the most common, low molecular weight neurotransmitters and metabolites in brain regions [84]. Using this method, 17 analytes were separated and then detected within 8 min and the LODs were found to be 0.03–0.2 nM for monoamine neurotransmitters, 0.05–11 nM for monoamine metabolites, 2–250 nM for amino acids, 0.5 nM for ACh, 2 nM for histamine, and 25 nM for adenosine at 5 μL sample volume. This method was successfully used to study the small brain regions by infusing the GABA receptor antagonist bicuculline (50 μM) into a rat ventral tegmental area while recording neurotransmitter concentration locally and in nucleus accumbens, revealed that complex GABAergic control over mesolimbic processes. In the period covered by this article, several interesting combinations and applications of LC-MS methods have been summarized in Table **2**. These results reveal that LC combined with MS techniques is an excellent platform for efficient separation of trace level of neurological substances in biological samples. Importantly, MS and tandem mass spectrometric techniques can be successfully applied to identify neurological substances and their metabolites, with a high grade of certainty, of minute amounts of substances contained in complex biological matrices. The LC-MS approaches also showed great potentiality to separate and detect the neurological drugs in biological samples with high sensitivity and high speed. Although the LC-MS techniques are effective for neurodrug analysis, they require specially designed columns, derivatization steps, and lengthy procedures for extraction and preconcentration of neurodrugs in biological samples [27, 44, 49, 63, 66]. 

## GAS CHROMATOGRAPHY-MASS SPECTROMETRY

GC-MS is a powerful technique in routine analytical laboratories by increasing sample throughput and improving laboratory efficiency [85]. It has excellent capability to separate and to detect volatile compounds including neurological chemical substances at trace levels in various samples. AM and MA are chiral compounds whose S-(+) enantiomers exhibit five times greater pharmacologic potency than the corresponding R-(−) enantiomers. AM and MA are both widely used as psychostimulants, but both substances are also metabolites of several therapeutic drugs. Peter’s group developed GC-MS methods with NCI mode for the determination of AM, MA, MDOAM, MDOMAM, and MDOEAM enantiomers in oral fluid [86, 87]. The authors described the enantioseparation of amphetamine and its enantiomers and their identificaiton by using GC-MS. These methods are rapid, sensitive and reliable for the assay of AM, MA, MDOAM and MDOMAM in oral fluid samples. The applicability of these methods were demonstrated by analysing >50 authentic samples. Kim’s group described a quantitative method for the determination of allopregnanolone (5α, 3α-THP) and related neurosteroids in human cerebrospinal fluid and plasma by using GC/ECNCI/MS [88]. This method involves the conversion of neurosteroids into carboxymethoxime, pentafluorobenzyl and trimethylsilyl derivatives and their detection with NCI mode. The LOD is < pg level with the exception of progesterone and dihydroprogesterone. This method was successfully applied to analyze four neurosteroids, including androsterone, testosterone, 5α, 3α-THP, and pregnenolone in 1-2 ml of CSF. This method confirmed the levels of 5α, 3α-THP and pregnenolone in human CSF were higher than those of monkey CSF. The levels of androsterone and pregnenolone are very low in rat plasma. 

Yonamine and co-workers used SPME coupled with GC-FID for detection of THC, AM, MA, COC and ethanol in saliva [89]. Saliva samples were subjected to an initial headspace procedure for ethanol determination by GC-FID. The analytes were extracted by submersing of a polydimethylsiloxane fiber (100 µm) in the vial (20 min) and subsequently extracted by alkalinization of sample and analytes were detected by GC-MS with SIM mode. This method showed good linearity, recovery, intra- and inter-assay precision as well as limits of detection and quantification for all analytes. The same group described highly precise SPME combined with GC-MS for sensitive detection of COC, BZE and cocaethylene (transesterification product of the coingestion of COC with ethanol) in human hair samples [90]. The LOQ and LOD were 0.1 ng/mg and 0.5 ng/mg for all the compounds, respectively. Moreover, Musshoff’s group developed a method using SPE with enantioselective derivatization coupled with GC-MS for detecting DA and of dopamine-derived tetrahydro-isoquinoline alkaloids (R)/(S)-salsolinol and norsalsolinol in human brain samples [91]. This study revelaed that the regional distributions of (R)-SAL and (S)-SAL and NSAL are in large collective of human brain samples which is obtained by autopsy. Furthermore, the concentrations of DA, SAL and NSAL were decreased significantly with rising age, which may be consistent with the apoptotic effects of ageing. Therefore, this study can be served as a reference for other studies in humans concerning the etiology of alcoholism or other neurodegenerative diseases with the involvement of tetrahydroisoquinolines. Koren’s team utilized HS-SPME coupled with GC-MS for detecting three principle opiates (COD, MP, and AMP) in human hairs [92]. The extracted analytes were dried under N_2_, derivatized, and subjected to HS-SPME coupled with GC-MS for their identification. Dórea and co-workers developed an analytical method for detecting THC, CBD and CBN in human hairs by HS-SPME coupled with GC-MS/MS [93]. The authors studied various parameter such as pH, mass of hair, fiber type, extraction temperature, desorption time, ionic strength, pre-equilibrium time and extraction time for the efficient extraction of target analytes. This method showed excellent linearity in the range of 0.1–8.0 ng/mg, with *R^2^* >0.994. The LODs and LOQs were 0.007–0.031 ng/mg and 0.012–0.062 ng/mg, respectively. 

Chang’s team described the potential application of SPE coupled with GC-MS with multiple ionization mode approach for simultaneous monitoring of amphetamines (AM, MA, MDOAM, MDOMAM, MDOEAM), ketamine (KT, NK), and opiates (MP, COD, AMP) in hair testing for common drugs of abuse in Asia [94]. In this method, EI and NCI were used for the identification of target analytes in hair samples. The SPE was used for the extraction of analytes in hair samples, derivatized using heptafluorobutyric acid anhydride at 70 °C for 30 min, and the derivatives were analyzed by GC–MS with EI and NCI. The LODs were 0.03 ng/mg for AM, MA, MDOAM, MDOMAM, MDOEAM, 0.08 ng/mg for KT, NK, MOR, and 0.06 ng/mg COD and for AMP by GC-EI-MS. The LOD of GC/NCI-MS was much lower than GC/EI-MS analysis. The LOD were 30 pg/mg by GC/EI-MS and 2 pg/mg by GC/NCI-MS for AP and MDA, respectively. The sensitivity of GC/NCI-MS was improved for 5-folds for AM and MDOMA than EI. Moreover, the sensitivity of GC-NCI-MS was greately improved from 15- to 60-folds for AM, MA, MDOAM, MDOMAM, MDOEAM, MP, and COD than EI. The integration of GC/EI-MS and GC/NCI-MS provided high sensitivity for abuse drug analysis in hair samples. 

Ishii *et al*., developed a new GC coupled with surface ionization organic mass spectrometric method for high sensitive measurements of PCP in body fluids [95]. This new technique showed good linearity (0.25−10 ng/mL) for quantification of analyte in whole blood or urine. The LODs (S/N= 3) were 0.05 ng/mL and 0.01 ng/mL for PCP in whole blood and urine, respectively. Macchia’s group reported the first application of HS-SPME coupled with GC-MS with PCI technique for simultaneous detection of MDOAM, MDOMAM, MDOEAM and MBDB in hairs [96]. This method provided high precision for both intra- and inter-day analysis with 2% and 10%, respectively. The LODs and LOQs were <0.7 and 1.90 ng/mg for each analyte, respectively. This method was successfully applied to analyze target species (AM, MA, KT, EP, NIC, PCP, MD) in hair and saliva samples of young people who participated in disco in the Rome area. Stout’s group performed a comparison study between LC-MS-MS and GC-MS for the monitoring of drugs (AM, MA, (±)-3,4-MDOAM, (±)-3,4-MDOMAM, (±)-3,4-MDOEAM, PCP, and (±)-THCCOOH) in blood [97]. This comparative study revealed that GC–MS and LC–MS–MS agreement results in AM, MA, MDOAM, MDOMAM, MDOEAM, PCP, and THCCOOH analysis and both techniques can provide accurate, precise, and specific results without interferences from structural analogues. Moreover, Hsu’s team developed a GC-MS method for PCP analysis in drug-abuse urine samples [98]. This method showed good repeatability and reproducibility with RSD values 2.1%-3.6% and 4.2%-7.3%, respectively. 

Nowadays, it is very important to determine the trace amounts of drugs and their metabolites in drugs-abuse samples. Marin’s group described a new method for determinations of THCCOOH and 11-OH-THC by using two-dimensional GC–MS [99]. This method was successfully validated for the confirmation of cannabinoids in 70 spiked samples prepared in drug-free meconium and in 46 residual patient specimens. Using this new 2D GC method, trace amount of THCCOOH was identified in 10 drug-abuse samples. The effective separation was observed between target analytes with reduced interferences by using 2D GC–MS. Staub *et al*. developed a rapid GC/NCI-MS/MS for the identification of THC, 11-OH-THC and THCCOOH in whole blood in 15min [100]. The LLE method was used to extract the cannabinoids from whole blood and then derivatized with trifluoroacetic anhydride and hexafluoro-2-propanol as fluorinated agents. Huestis’s team described a SPE coupled with 2D-GC-EI-MS method for the simultaneous identification and quantification of THC, CBD, CBN, and metabolites 11-OH-THC and THCCOOH in oral fluid [101]. This method allows to detect five cannabinoids at pg/mL in ora fluid. In this method, SPE was performed by using CEREX® Polycrom™ THC SPE columns. Analytes were eluted (THC, 11-OH-THC, CBD, and CBN) with hexane/acetone/ethyl acetate (60:30:20, *v/v/v*), derivatized with *N,O-bis*-(trimethylsilyl)trifluoroacetamide and then identified by 2D-GC-MS with cold trapping. Importantly, THCCOOH was separately eluted with hexane/ethyl acetate/acetic acid (75:25:2.5, *v/v/v*), derivatized with trifluoroacetic anhydride and hexafluoro-isopropanol, and quantified by 2D-GCMS with NCI. This method showed good linearity in the range of 0.5–50 ng/mL for THC, 11-OH-THC, CBD and 1–50 ng/mL for CBN. 

Dórea and co-workers developed a new method for determination of THC, CBD and CBN in human hair samples by using HF-LPME coupled with GC-MS/MS [102]. This method involves the extraction of cannabinoids from human hair samples using HF-LPME with a 6 cm polypropylene fiber (600 μm i.d., 200 μm wall thickness, 0.2 μm pore size). Various extraction parameters such as type of extraction solvent, pH, stirring speed, extraction time, and acceptor phase volume for efficient extraction of cannabinoids form hair samples were optimized. The best extraction (10 mg hair sample) was achieved by using flowing parameters; 20 μL of butyl acetate; aqueous (pH 14) donor phase containing 6.8% NaCl; 600 rpm stirring speed; 20 min extraction time. The separated analytes were identified by GC-MS/MS. This method showed good linearity (1 – 500 pg/mg) with *R^2^* >0.99. The LODs and LOQs were 0.5–15 pg/mg and 1–20 pg/mg, respectively. This method was successfully applied to identify the cannabinoids (CBD, THC and CBN) in hair samples from patients in a drug dependency rehabilitation center. Recently, Andrews and Paterson described the potentiality of LLE coupled with 2D-GC-MS method for the identification and quantification of THC, CBD, CBN, 11-OH-THC and THCCOOH in post-mortem blood specimens [103]. The extracted analytes were derivatized with *N*-methyl-*N*-(trimethylsilyl)trifluoroacetamide and then separated and identified by 2D-GC-MS. The LODs were 0.25 ng/mL for all analytes. The LOQs were 0.25 ng/mL for THC, CBN, 11-OH-THC and 0.5 ng/mL for CBD and THCCOOH, respectively. The assays had a linear range (0.25–50 ng/mL) with *R^2^* ≥0.992 for all analytes. This method is well suited for the analysis of cannabinoids in 54 post-mortem blood specimens. 

Nicotine in hair was referred as a biomarker for monitoring long-term environmental tobacco smoke exposure and smoking status. GC-MS was used for nicotine analysis in hair samples and it is a high throughput methodology for the extraction and identification of nicotine with 100 hair samples per day [104] with good linearity with *R^2^* > 0.995. Nicotine is a major addictive compound in cigarette and its smoke is rapidly and extensively metabolized to form several metabolites in human. Cotinine is a major metabolite of nicotine and used as a biomarker to detect smokers. Man’s team developed a simple, sensitive, rapid and high throughput GC–MS method simultaneous quantification of urinary NIC and COT in passive and active smokers [105]. LLE was used to extract NIC and COT, and the obtained extract was directly injected into GC-MS for analysis. This method showed good linearity (0.5–5000 ng/mL) with *R^2^* >0.997. The LODs were found to be 0.20 ng/mL for NIC and COT and the average recoveries of NIC and COT were 93.0 and 100.4%, respectively. This method was successfully applied to determine NIC and COT in the samples for smokers and non-smokers. An analytical method has been developed for the simultaneously determination of NIC, COT, NC, and trans-OH-COT in human oral fluid [106]. The analytes were extracted by SPE and then detected by GC-EI-MS with selected ion monitoring mode. The average recoveries were 90–115% NIC, 76–117% COT, 88–101% NC, and 67–77% *trans*-OH-COT, respectively. Using this method, nicotine and three metabolites were effectively analyzed in oral fluid of smokers with high precision and accuracy. Recently, Khorrami and Rashidpur developed a molecular sol–gel imprinting approach for selective and direct immersion of SPME coupled with GC-MS for sensitive detection of caffeine in human serum [107]. SPME was performed by using polymerization mixture (vinyl trimethoxysilane and methacrylic acid as vinyl sol–gel precursor) and functional monomer. The fiber was directly injected into GC-MS for caffeine analysis from biological samples. Various extraction parameters such as extraction time, temperature and stirring speed were studied and the LOD was 0.1 μg/mL. An overview of GC-MS methods for analysis of neurochemicals and their metabolites in biological samples is provided in Table **3**. These GC-MS based techniques allow rapid separation and detection of neurological drugs in various samples. However, these methods require tedious derivatizations steps and sample handling [88, 92, 100-101]. Importantly, EI and CI ionization methods are suitable well to couple with GC technique and applied for volatile compound analysis. Although GC-MS allows rapid analysis of many analytes, alternative ionization techniques are required to be developed for the analysis of the majority of neurodrugs in the future.

## CAPILLARY ELECTROPHORESIS-MASS SPECTRO-METRY 

Capillary electrophoresis is a very useful separation technique for determining neurological substances and their metabolites in body fluids because of its advantages such as high speed, higher resolution, and a smaller injection volume than HPLC or GC. Although many detectors are used in CE, high sensitivity detection of neurodrugs and their metabolites in biological, clinical and forensic samples at ultra-trace level only can be achieved by CE combined with mass spectrometric techniques [108-111]. Recent developments on CE-MS methods for analysis of neurological substances in various samples are discussed below.

Catecholamines are neurotransmitters in the central and peripheral sympathetic nervous system. The diagnosis of diseases like Parkinsonism is required to determine catecholamines and their metabolites in biological samples. Siren’s group described the application of CE combined with ESI-MS for the analysis of catecholamines in urine samples with LODs 0.5 - 1.3 µM [112]. The electroosmotic mobilities of catecholamines were decreased from water to 1-propanol, and correlated with the dielectric constants of the solvents and the optimal solvent is ethanol. Nilsson’s and co-workers described the use of CE combined with APPI- and ESI- mass spectrometric techniques for 11 pharmaceutical drug analysis using potassium phosphate and AF buffers [113]. Compared with ESI, the APPI is superior on cluster-free background. APPI is less affected by non-volatile salts in the CE buffers. Sasijima’s group described the non-aqueous CE with ESI-TOF mass spectrometric method for simultaneous determination of 20 antidepressants in plasma samples [114]. In this method, AA buffer (60 mM) and acetic acid (1 M) in ACN, water and MeOH (100:1:0.5, *v/v/v*) were used as background electrolyte. The target analytes were accurately detected with reduced background noise by using TOF-MS. SPE was used for the extraction of antidepressants from plasma. The LODs and LOQs were 0.5–1 and 1–5 ng/mL for all analytes. 

Morphine and codeine are opioids and are regarded as the benchmark of opioid analgesics to relieve from severe or agonizing pain and suffering. Therefore, their identification is important in the biological, forensic toxicology, pharmacokinetic and pharmacogentic research. In this area, GC-MS and LC-MS methods are widely used for the identification of neurological substances in biocomplex samples. However, CE coupled with mass spectrometry has also proven to be a promising tool for sensitive analysis of neurocompounds in clinical and forensic samples. For example, Tagliaro’s team reported the potential applications of CE-MS for the identification of illicit drugs [115-117]. Briefly, CZE-ESI-TOF-MS was used for the efficient separation and identification of drugs (MDOAM, MDOMAM, MD, COC, MP, COD and AMP) in hair samples [115]. The effect of buffers such as phosphate, borate and Tris buffers were investigated as the optimal buffer to achieve the ideal separation and the best mass spectra for these drugs. Among these, the ammonium phosphate buffer was the best choice. But, inorganic non-volatile borate and Tris buffers are found to be hardly suitable for the separation and identification of above analytes by CE-MS. The same group described the application of CZE-ESI-TOF-MS method for the identification of illicit and abused drugs (MA, MDOAM, MDOEAM, MDOMAM, MD, COC, MP, COD, AMP, BZE) in blood [116]. These analytes were separated with capillary zone electrophoresis by applying 15 kV within 25 min, in an uncoated fused-silica capillary (75 microm x 100 cm) using AF electrolyte solution (25 mM, pH 9.5). The capillary electropherograph was connected with TOF-MS through an orthogonal electrospray ionization source, with a coaxial sheath liquid interface for their identification. In this method, isopropanol-water (1:1 v/v) contained 0.5% formic acid solvent system was used as the sheath liquid. Using this method, good linearity (10-2000 ng/mL) was observed with *R^2^* 0.9744 – 0.9982 for all analytes. The LODs were in the range of 2-10 ng/mL (S/N > or =3) and LOQs were 10-30 ng/mL for all analytes. Moreover, a simple and rapid CZE–MS method was developed for the sensitive and quantitative determination of drugs and their metabolites (AMP, MP, AM, MA, MDOAM, MDOEAM, MDOMAM, BZE, EP and COC) in drugs-abuse human hair [117]. LLE method was used for the extraction of drugs from hair samples. In this method, CZE separations were carried out in a 100 cm ×75 μm (I.D.) uncoated fused silica capillary. The best separation was achieved by using AF buffer (25 mM, pH 9.5) with separation voltage 15 kV. The ESI-MS was performed with positive ion mode by using the following conditions: capillary voltage 4 kV, nebulizer gas (nitrogen) pressure 3 psi, source temperature 150 °C and drying gas (nitrogen) flow rate 8 l/min. In this study, isopropanol–water (50:50, *v/v*) with 0.5% formic acid system was used as sheath liquid and the ion trap MS operated in a selected ion monitoring mode for each drug/metabolite. Under the optimal conditions, all the compounds were separated within 20 min and the LODs were <0.1 ng/mg. This method showed good precision with RSDs ≤3.06% for migration times and ≤22.47% for areas in real samples, in both intra-day and day-to-day experiments. 

Oxycodone and its metabolites (OMOR, NOCOD and NOMOR) belong to opioids that contains OH group at position 14. Wey and Thormann described the use of capillary electrophoresis and capillary electrophoresis-ion trap multiple -stage mass spectrometric techniques for the differentiation and identification of OMOR, NOCOD and NOMOR in human urine [118]. Using a binary phosphate buffer containing 60% ethylene glycol (pH 7.9), the migration order of OCOD and OMOR with respect to their *N*-demethylated analogs was found to be reversed compared to that observed for codeine, dihydrocodeine, morphine and dihydromorphine, compounds that do not have an OH group at position 14. The optimum separation was achieved at pH 9.0. In this study, OCOD, OMOR, NOCOD and NOMOR were used as reference compounds, since opoids contains OH group at position 14. In comparison with their MS^2^ and MS^3^ data, it was interestingly noticed that all tested compounds lost H_2_O at the MS^2^ level, i.e. after isolation and fragmentation of [M+H]^+^ ions. These results revealed that the neutral loss seemed to be a characteristic behavior for opioid structures carrying an OH group at position 14 of the molecule, which was also confirmed structurally in antagonist naloxone. The same group developed a head-column field-amplified sample stacking CE-ESI-MS/MS method for the sensitive identification of opioids in urine [119]. The SPE and LLE sample pretreatments were used in between for the extraction of analytes prior to their separation and identification by CE-ESI-MS/MS. This method was successfully applied to separate and to detect ultra-trace amounts of target analytes with 1000-folds enhanced sensitivity. Meanwhile, Clench’s group described the application of SPE coupled with CE-MS technique for the identification of nicotine and 8 of its metabolites in urine [120]. The recoveries were found to be 98%. The LODs were 0.11 and 2.25 μg/mL for NIC and COT, respectively. 

Sweedler’s group developed a single-cell mass spectrometric method for the rapid identification of endogenous compounds in over 50 identified and isolated large neurons from the *Aplysia californica* central nervous system [121]. The single-cell CE system coupled with ESI-MS was used for simultaneous measurement of a vast array of endogenous compounds in over 50 identified and isolated large neurons from the *Aplysia californica* central nervous system. Using this method, more than 300 distinct ion signals (*m/z* values) were detected from a single neuron in the positive ion mode, 140 of which were selected for chemometric data analysis. This method also evaluated the metabolic features of 6 different neuron types (B1, B2, left pleural 1 (LPl1), metacerebral cell, R2, and R15) and their various physiological functions. Moreover, the metabolite content of individual neurons (80, 300 μm in diameter) was assessed for these 6 genotypes and their chemical differences were studied. The results indicated that the chemical similarities among some neuron types (B1 to B2 and LPl1 to R2) and distinctive features for others (MCC and R15 cells). Meanwhile, acetylcholine has registered high ion counts in both the R2 and LPl1 neurons. In contrast, MCC cells contain serotonin, an observation confirmed by independent studies reporting that MCC neurons use serotonin as a neurotransmitter. Interestingly, a unknown mass speak was observed at *m/z* 267.1 (1 ppm accuracy) in the R15 neurons which correspond to the dipeptide ThrPhe (or PheThr). Principal component analysis of CE-ESI-MS results revealed that the neurons are exhibited individual as well as phenotypic differences in their chemistry and neuron pairs possessed similar chemical compositions. This CE-ESI-MS platform allows an automated neuronal sampling (with less dilution and perhaps greater automation) for the small-molecule analysis in central nervous system. 

The same group described the application of CE-ESI-TOF-MS for the analysis of metabolomic profiling in single cells and subcellular structures [122]. In this method, water/MeOH/AcOH (49/50/1, *v/v/v*) used as a solvent for the metabolite extraction in each cell. Since, it also served as a protonating agent to protonate chargeable metabolites, thereby facilitating CE separation and ESI-MS detection. The mass peak at *m/z* 177 corresponds to serotonin in metacerebral cell neuron, which is due to the addition of 7.5 µM of standard 5-HT. These results revealed that the high degree of similarity between the MCC sample and the 5-HT standard which confirms the presence of 5-HT in the neuron. The presence of ACh was confirmed by MS/MS and yielded a fragment ion at *m/z* 87 which consistent with the fragmentation profile of ACh. The LODs were <50 nM (<300 amol) for a number of cell-to-cell signaling molecules, including ACh, histamine, DA, and serotonin. This technique was successfully applied to identify neurons in single cell metabolomic profiling in *Aplysia californica*-the R2 neuron and metacerebral cell. The neurotransmitters were detected within the cells (ACh in R2 and serotonin in MCC) and had molecular masses consistented with all of the naturally-occurring amino acids (except cysteine). The MS/MS was used to distinguish ACh from isobaric compounds in the R2 neuron and identified the single cell metabolites. These analytical platforms are well-defined functional networks that enable single cell measurements as well as changes in the cellular metabolome to be related to cell function. Typical examples of CE-MS methods for the analysis of neurological substances in biological samples are summarized in Table **4**. These approaches facilitate the rapid and accurate determination of neurological compounds in biological samples by ESI- and MALDI- MS with minimal volumes of samples. Although it is capable to analyze drugs with minimal volume of sample, it required tedious sample cleaning procedures, lengthy derivatization procedures, unstable coating layers beyond a limited pH range, and poor reproducibility [115-118]. Therefore, the technical difficulties in linking micro and capillary separation techniques with nanospray MS are needed to be solved and more advancements can be expected in the future.

## DIRECT MASS SPECTROMETRIC TECHNIQUES

**Fig. (1):**
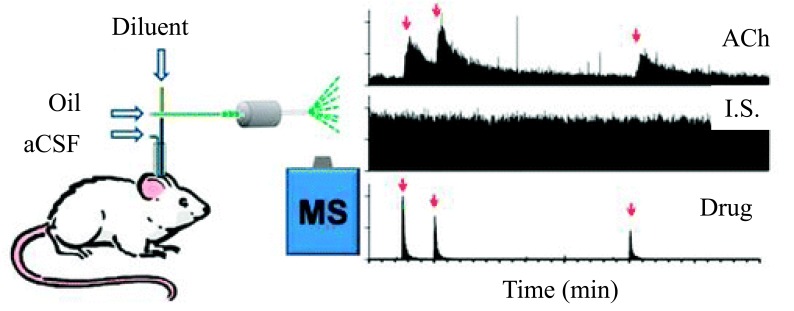
Microdialysis sampling was coupled with segmented flow ESI-MS to monitor acetylcholine in the brain of live rat. Adapted from Reference [123].

The development of sensors-based analytical methods for *in vivo* monitoring of neurochemicals is important in neuroscience because it allows correlation of neuro-transmission with behavior, disease state, and drug concentrations in the intact brain. Therefore, Kennedy’s group described a novel approach for the monitoring of neurochemicals using microdialysis sampling coupled with segmented flow ESI-MS [123]. In this method, segmented flow permits to prevent Taylor dispersion of collected zones and allows to rapidly identifying the target analytes (5 s) by using direct ESI-MS with temporal resolution. The MS-based “sensor” was successfully used to monitor ACh in the brain of live rats (Fig. **1**). The LOD is 5 nM for ACh, which is sufficient to monitor basal ACh as well as dynamic changes elicited by microinjection of neostigmine (an inhibitor of acetycholinesterase), which evoked rapid increases in ACh and tetrodotoxin (a blocker of Na^+^ channels), which lowered the ACh concentration. This method was used as a sensor for the simultaneously monitoring of neurodrugs, metabolites and infused drugs *in vivo*. 

Streibel and co-workers developed a novel microprobe sampling device (μ-probe) for *in situ* on-line photo ionization mass spectrometric method for the analysis of volatile chemical species formed within objects consisting of organic matter during thermal processing [124]. In this method, occurring of the chemical signatures were investigated in cigarette during heating, pyrolysis, combustion, roasting and charring of organic material within burning objects such as burning fuel particles (e.g., biomass or coal pieces), lit cigarettes or thermally processed food products (e.g., roasting of coffee beans). For these identifications, the tip of the μ-probe is inserted directly into the tobacco rod and volatile organic compounds of the burning cigarette are extracted and then connected to μ-probe sampling tool combined with photo ionization time-of-flight mass spectrometry for the real-time analysis of organic compounds in burning cigarette. This method provided chemical structures in burning Kentucky 2R4F reference cigarette and studied behaviors of nicotine, and a range of semi-volatile aromatic and aliphatic species in burning cigarette. It was noticed that the homologous series of alkylated polycyclic aromatic hydrocarbons and fatty acids can be traced to higher masses and more aromatic and carboxylic acid compounds can be identified in mass spectrum. At the same time, nicotine (*m/z* 162) and nicotine fragment (*m/z* 84) are also identified by using 126 nm photons. Furthermore, several other compounds are also detected at *m/z* 43 (carbohydrate fragement), 44 (acetaldehyde), 58 (acetone), 79 (pyridine), 98 (furfuryl alcohol), 110 (dihydroxybenzenes) and 194 (caffeine), respectively. Therefore, μ-probe sampling coupled with soft photo-ionisation mass spectrometry is a promising tool for the analysis of chemical signatures in pyrolysis and combustion gases in a burning cigarette as well as inside a coffee bean.

Hopfgartner *et al*. described the MALDI-TOF mass spectrometric method for the qualitative and quantitative analysis of erlotinib (RO0508231) and its metabolites in rat tissue sections from liver, spleen and muscle [125]. In this method, sinapic acid was used as the matrix for the analysis of target analytes by MALDI MS. LC-ESI-MS/MS was also used for the drug quantitation in tissue extracts by the standard addition method. Using this method, the parent compound and its O-demethylated metabolites were confirmed in all tissue types and calculated their absolute amounts in tissue samples. It was found that liver contains intact drug at 3.76 ng/mg, while in spleen and muscle 6- and 30-fold lower values, respectively. These results were compared with drug quantitation obtained by whole-body autoradiography and found to be similar. Furthermore, MALDI- imaging MS allows to detect/measure the target compounds in liver and spleen tissues. Recently, Andren’s group synthesized deuterated matrix namely D^4^-α-cyano-4-hydroxycinnamic acid (D^4^-CHCA) and used as a matrix for matrix-assisted laser desorption ionization-mass spectrometry (MALDI-MS) and MALDI-MS imaging (MSI) of small molecule drugs and endogenous compounds [126]. The cluster and fragment peaks of CHCA are shifted to +4, +8 and +12 Da, which expose signals across areas of the previously concealed low mass range. Using this technique, obscured MALDI-MS signals of target analytes (pharmaceutical small molecule, isoquinoline, and endogenous compounds including the neurotransmitter ACh) have been unmasked and visualized directly in biological tissue sections. Furthermore, Shin’s team described the potential application of MALDI imaging MS for analysis of small molecule pharmaceutical compounds directly on tissue sections to determine spatial distribution within target tissue and organs [127]. In this method, LC-ESI-MS/MS was used for the absolute quantification of target analytes in tissues. Using MALDI MS, loperamide (an antidiarrheal agent) and a P-glycoprotein substrate were successfully quantified in mouse brain tissue sections. This method showed good linearity in the range of 0.025 - 0.5 μM with *R²* 0.9989. The MALDI mass spectrum showed loperamide mass peak at *m/z* 477 and observed the predominant product ion at *m/z* 266 which confirmed the cleavage at adjacent to the piperidine ring of loperamide (477→266). This method was successfully used to demonstrate the direct quantification of loperamide in tissue sections with high sensitivity. 

## CONCLUSIONS AND PERSPECTIVES

Recently, mass spectrometry has matured as a versatile, high-performance technique for sensitive analysis of neurological substances in biological and drug-abuse samples. The reported approaches effectively demonstrated for the detection of neurological substances with high sensitivity from the complex matrices by using extraction methods (LLE, HS-SPME, SPME, SPE) coupled with LC-, GC- and CE-MS. In addition, a series of novel mass spectrometric tools have been developed to monitor neurological substances *in vivo *in various organs. Selectivity, sensitivity and efficiency of MS methods were greatly improved by either employing new separation mechanisms or developing new ionization methods. The combination of sample pretreatment methods coupled with chromatographic methods- (LC, GC and CE) MS techniques permits sensitive analysis of neurocompounds in neuroscience, especially when the analytes are present at trace levels. The neurological substances were confirmed by tandem MS and distinguished the neurochemcials in neurons and identified the neuro-transmitters and metabolites in single cells. These MS- based analytical platforms are well-defined and identified the functional networks that enable single cell measurements in neurons. Importantly, minimal sample volumes were consumed for the analysis of trace amounts of neurological substances in biocomplex samples and *in vivo*. Finally, together with the sample pretreatment and chromatographic techniques combined with MS approaches have been proved as versatile tools for solving neuroscience analytical questions far beyond the routine task of neurological substances analysis in biolgocial tissues. Advanced mass spectrometric trchniques have become effective platforms for drug analysis, and this role should be more significant to the medical world in the near future.

## Figures and Tables

**Table 1.** Classification of Neurodrugs and their Generic Names, Molecular Weights, Structures and Trade Names

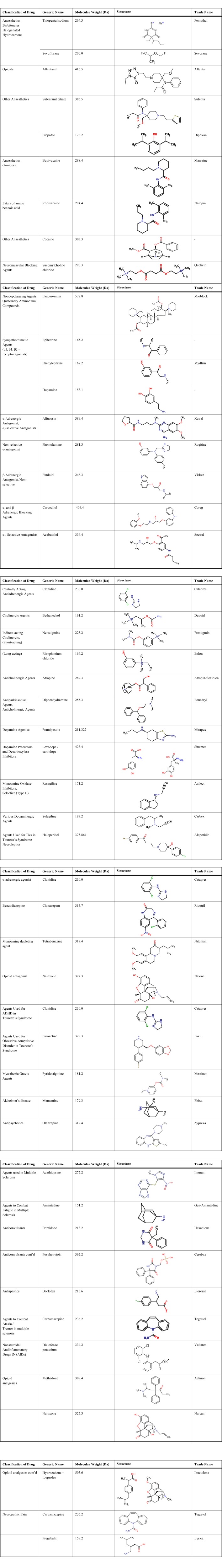

**Table 2.** LC – MS Methods for the Identification of Neurochemicals and their Metabolites in Various Samples

Name of Neurochemicals	Matrix	Sample Preparation	Chromatographic Technique	Detection Technique	Limit of Detection	Refs.
DMI, IM, NOR, AMT, CL	Plasma	SDME	LC- AA buffer (0.01 mM, pH 5.50) : ACN (50:50, *v/v*)	ESI-MS	0.1^a^	[19]
AM, MA	Plasma	SDME	LC- ACN-water	ESI-MS/MS	0.3 - 0.04^b^	[20]
FLU, PXT, SRT, FLV, CIT, MIL, VEN, MIR, NF, DMCTP, DDMCTP, DMVEN, DMMIR	Blood	LLE	LC - ACN/AF buffer (4 mM, pH 3.2)	ESI-MS/MS	10 b	[21]
AMT, CTP, CL, DMI, DMCTP, DMCL, DMDS, DMD, DMFLU, DMVEN, DDMCTP, DOS, DOX, FLU, FLV, IM, MAT, MIA, MIR, MOC, NOR, PXT, RBX, SRT, TRZ, VEN	Plasma	LLE	LC	ESI-MS/MS	LOQ: 2.5 -10^a^ LOD: 0.2 - 10^a^	[22]
AMT, NOR	Rat plasma	LLE	LC - AA(0.6% formic acid)-ACN (60:40, *v/v*)	ESI-MS	~1.0^a^	[23]
FLU, CTP, PXT, VEN	Plasma	SPE	LC- water (formic acid 0.6 mM- AA: 30 mM)-ACN (35:65, *v/v*)	ESI-MS	0.1 - 0.5^a^	[24]
VEN and its three metabolites ODV, NDV DDV	Human plasma	LLE	LC- water (AA-30mM, formic acid- 2.6 mM and TFA-0.13 mM) and ACN (60:40, *v/v*)	ESI-MS	0.2 - 0.4^a^	[25]
VEN and its metabolite ODV	Plasma	-	UPLC- AA-30 mM -MeOH (15:85, pH 6.0)	ESI-MS	1.0^a^	[26]
VEN, ODV	Plasma	LLE	LC - AA (10 mM) and MeOH	ESI-MS	LOQ: 0.2^a^	[27]
ENP, ENPT	Plasma	LLE	LC- MeOH-water-formic acid (62:38:0.2, *v/v/v*).	ESI-MS/MS	LOQ: 0.63^a^	[28]
ENP, ENPT	Plasma	LLE	LC	ESI-MS/MS	LOQ: 0.1^a^	[29]
ENP, ENPT	Plasma	SPE	LC	ESI-MS/MS	LOQ: 0.2, 1.0^a^	[30]
ENP, ENPT	Plasma	SPE	LC	ESI-MS/MS	0.064^a^	[31]
ACh, Ch, iso-ACh	Rat brain	Microdialysis	LC-IC- AF, AA and ACN	ESI-MS/MS	0.2 - 0.6^c^	[32]
ACh, Ch, acetyl-β-methylcholine	Rat brain dialysates	Microdialysis	LC-IC	ESI-MS/MS	0.05, 3.75^c^	[33]
ACh	Rats	Microdialysis	HILIC	ESI-MS/MS	0.075^c^	[34]
ACh	Rat brain	Microdialysis	LC-2% of ACN and 0.05% of TFA	APCI-MS/MS	LOQ:0.15^d^	[35]
[Met]enkephalin and [Leu]enkephalin	Freely-moving rats.	Microdialysis	LC-	ESI-MS/MS	0.001, 0.002^d^	[36]
Endogenous ACh	Rodent brain	Microdialysis	LC-	ESI-MS/MS	0.04^d^	[37]
Endogenous opioid peptides	Rat striatum	Microdialysis	LC	ESI-MS/MS	5.0^d^	[38]
DA, 5-HT, NE	-	Microdialysis	LC	ESI-MS/MS	0.2, 1.0, 0.9^d^	[39]
GABA, Glu	Brain	Microdialysis	HILIC - 0.1% formic acid in water and ACN	ESI-MS/MS	1.0^d^	[40]
ACh, Ch	Rat and mouse brain	Microdialysis	LC- AF (20 mM, pH 3.3) and ACN (20:80)	ESI-MS/MS	&emsp;0.02, 10^c^	[41]
ACh	Rat brain	Microdialysis	LC	ESI-MS/MS	1.4^c^	[42]
NMN, MN, 3-MT	Plasma	SPE	LC	ESI-MS/MS	< 0.1^d^	[43]
MN, NMN	Plasma	SPE	LC	ESI-MS/MS	-	[44]
MN, NMN	Urine	SPE	LC	ESI-MS/MS	2.5^d^	[45]
5-HT, and its precursors, 5-HTP, TP	Chocolate samples	Acidic extraction	cLC-ACN and AF (5 mM, pH 4, 3:97, *v/v*)	ESI-MS/MS	0.01, 0.11^e^	[46]
5-HT, MEL, trans- and cis-piceid, and trans- and cis-resveratrol	Fruits	LLE	HPLC- ACN and water with 0.1% formic acid	ESI-MS	-	[47]
5-HT	Plasma	LLE	LC-MeOH	ESI-MS/MS	1.5^d^	[48]
CTP, FLU, FLV, PXT, SRT, and their main active metabolites DMCTP, NF	Plasma	LLE and SPE	LC- AA (50 mM, pH 8.1) and ACN	ESI-MS/MS	-	[49]
Glu, GABA, Ch, ACh, DA, 5-HIAA, 5-HT, DOPAC, HVA	Rat brain	-	UHPLC	ESI-MS/MS	LOQ: 2.440^e^	[50]
5-HT, DA, 5-HIAA	Urine	-	-	nano-ESI-MSn	9 - 12.9^d^	[51]
GLUs, 5-HT, DA, 5-HIAA, DOPAC, HVA	Rat brain	Microdialysis	LC	ESI-MS/MS	-	[52]
DA and its phase I and phase II metabolites	Rat and mouse brains	Microdialysis	LC	ESI-MS/MS	0.8^d^	[53]
Neuroleptics clozapine, flupentixol, haloperidol, penfluridol, thioridazine, and zuclopenthixol	Hair samples	SPE	LC - Solvent A, (AF (1 mM)/0.1% formic acid, pH 3) and solvent B (ACN/0.1% formic acid)	ESI-MS/MS	0.05^e^	[54]
MP, MAM, COD, COC, BZE	Hair	SPE	-	ESI-MS/MS	0.004 -0.039^e^	[56]
MD, EDDP, COC BZE, AMP, MP, COD	Human umbilical cord	SPE	LC	ESI-MS	<0.0025^e^	[57]
MP, COD, AMP, EME, BZE, COC, CCE, AM, MA, MDOAM, MDOMAM, MDOEAM	Blood	SPE	LC- AF (20 mM, pH 2.8) (solvent A) and ACN/solvent A (90:10, *v/v*) gradient	ESI-MS/MS	-	[58]
LSD, iso-LSD and the metabolite 2-oxo-3-hydroxy-LSD	blood	LLE	LC	ESI-MS/MS	LOQ: 0.01^e^	[60]
LSD and iso-LSD	Blood and urine	LLE	LC	ESI-MS/MS	LOQ: 0.02^b^	[61]
PCP	Oral fluid	SPE	LC	APCI-MS/MS	LOQ; 5^a^	[62]
PCP, AM, MC, MA, MDOAM, MDOEAM, MDOMAM, COC, BZE, KT, PSC	Oral fluid	MSPE	LC	ESI-MS/MS	LOQ: &emsp;0.3 - 4.9^a^	[63]
THC, 11-OH-THC, THCCOOH, CBN, CBD, THC-GLU, THCCOOH-GLU	Urine	LLE	LC- AA (10 mM, pH 6.15) and 15% MeOH in ACN	ESI-MS/MS	-	[64]
CB substances (CP 47, 497)	Urine	LLE	LC-	ESI-MS/MS	10^a^	[65]
THC, 11-OH-THC, THCCOOH, CBN, CBD	Blood	LLE	LC- water and ACN, both containing 0.2% formic acid	ESI-MS/MS	0.25 - 0.80^a^	[66]
NIC, OH-COT, CNO, CAF, ARC	Breast milk	LLE	LC- AF (50mM, pH 5.0) and ACN	ESI-MS/MS	1.6 - 16.0^b^	[69]
NIC, COT, trans-OHCOT	Meconium samples	SPE	LC	ESI-MS/MS	-	[70]
NIC, COT, trans-OHCOT, NC	Human plasma	-	LC	ESI-MS/MS	-	[71]
NIC-GLU, CNO, trans-OH-COT, NC, trans-NICO, COT, NN, NIC, AT, AB, COT-GLU	Human plasma and urine	SPE	LC	ESI-MS/MS	1.0 - 2.5^a^	[74]
NIC, COT, trans-OH-COT, VCL	Plasma	SPE	LC - AA (10 mM, pH 3)/ACN	ESI-MS/MS	< ng/mL	[75]
NIC, COT, trans-OH-COT, NNO, CNO	Urine	LLE	LC	ESI-MS/MS	LOQ: 10^a^	[76]
Nicotine-N-glucuronidation	Liver	LLE	LC	ESI-MS/MS	LOQ: 10^d^	[78]
NIC, COT, NN, NC, NIC-GLU, COT-GLU, NNO, CNO, trans-OH-COT, AB, AT	Rat brain	SPE	LC	ESI-MS/MS	< 0.025^e^	[80]
NIC, NN, AT, AB, COT	Urine	SPME	LC- AF (5 mM)/MeOH (55/45, *v/v*)	ESI-MS/MS	0.015-0.040^b^	[81]
NIC, COT and their metabolites	Urine	-	UPLC	ESI-MS/MS	0.7, 0.4^b^	[82]
NIC and its metabolites	Dog hair	LLE	IC	FT-MS	0.05- 0.23^e^	[83]
Neurochemicals	Brain	-	HPLC	ESI-MS/MS	0.05 -250^d^	[84]

a ng/mL;

b µg/L;

c fM;

d nM;

e ng/mg.

**Table 3.** An Overview of GC-MS Methods for the Analysis of Neurochemicals in Biological Samples

Name of the Neurological Drug	Matrix	Sample Preparation	Chromatographic Technique	Detection Technique	Limit of Detection	Ref.
AM, MA	Blood	SPE	GC	CI	1.0^b^	[86]
AM, MA, MDOAM, MDOMAM, MDOEAM	Oral fluid	-	GC	CI	-	[87]
Allopregnanolone (5α,3α-THP) and related neurosteroids	CSF and plasma	SPE	GC	ECNCI/MS	<pg/mL	[88]
THC, AM, MA, COC, EtOH	Saliva	SPME	GC	FID	LOQ: 0.01 - 5.0a	[89]
COC, BZE, CCE	Human hair	SPME	GC	FID	0.1^c^	[90]
DA, (R)/(S)- SAL, NSAL	Human brain	SPE	GC	-	-	[91]
COD, MP, AMP	Human hair	HS-SPME	GC	-	LOQ: 0.005 -0.01^c^	[92]
THC, CBD, CBN	Human hair	HS-SPME	GC	-	0.007-0.031^c^	[93]
AM, MA, MDOAM, MDOMAM, MDOEAM, KT, NK, MP, COD, AMP	Human hair	SPE	GC	EI and NCI	0.03 - 0.08^c^	[94]
PCP	Blood and urine	-	GC	SIOMS	0.05^a^	[95]
MDOAM, MDOMAM, MDOEAM, MBDB	Hair	HS-SPME	GC	PCI	<0.7^c^	[96]
PCP	Blood and urine	SPE	GC	-	-	[98]
THC, 11-OH-THC, THCCOOH	Blood	LLE	GC	NCI-MS/MS	<0.5^a^	[100]
THC, CBD, CBN, 11-OH-THC, THCCOOH	Oral fluid	SPE	GC	2D-GCMS	< 7500^a^	[101]
THC, CBD, CBN	Human hair	HF-LPME	GC	MS/MS	500-15000^c^	[102]
THC, CBD, CBN, 11-OH-THC, THCCOOH	Blood	LLE	GC	MS/MS	0.25^a^	[103]
NIC	Hair	LLE	GC	MS	<0.04^c^	[104]
NIC, COT	Urine	LLE	GC	MS	0.20^a^	[105]
NIC, COT, NC, trans-OHCOT	Human oral fluid	SPE	GC	EI-MS	< 5^a^	[106]
CAF	Human serum	SPME	GC	MS	100^a^	[107]

a ng/mL;

b µg/L;

c ng/mg.

**Table 4.** CE-MS Methods for the Analysis of Neurological Chemicals in Biofluids

Name of the Neurological Drug	Matrix	Technique	LOD	Refs.
20 antidepressants	Plasma	SPE-CE-ESI-TOF-MS	0.5-1.0^a^	[114]
MA, MDOAM, MDOMAM, MDOEAM, MD, COC, MP, COD, AMP, BZD	Blood	LLE-CZE-ESI-TOF MS	2-10^a^	[116]
AMP, MP, AM, MDOAM, MDOMAM, MDOEAM, BZD, EP, COC	Hair	LLE- CZE-ESI-TOF MS	< 0.1^b^	[117]
OMOR, NOCOD, NOMOR	Urine	LLE-CE- ESI-TOF MS	10 - 300^a^	[118]
DHC, NDHC, DHM, NDHM, COD, NMP, NC, MP	Urine	LLE-CE- ESI-TOF MS	<pp^b^	[119]
NIC and its metabolites	Urine	SPE-CE-ESI-MS/MS	110 - 2250^a^	[120]
Neurotransmitters	CNS	CE-ESI-MS	-	[121]
ACh, histamine, DA, 5-HT	CNS	CE-ESI-MS	< 50^c^	[122]
ACh	Live rats brain	ESI-MS	5^c^	[123]
Volatile organic compounds	Cigarette	PI-TOFMS	-	[124]
Erlotinib (RO0508231) and its metabolites	Rat tissues	MALDI-MS	3.76^b^	[125]
ACh	Tissues	MALDI-MS	-	[126]
Loperamide	Mouse brain	MALDI-MS	25^c^	[127]

a ng/mL;

b ng/mg;

c nM.

## ABBREVIATIONS

AA =: ammonium acetate
AB =: anabasine
ACh =: acetylcholine
ACN =: acetonitrile
AF =: ammonium formate
AM =: amphetamine
AMP =: 6-acetylmorphine
AMPA =: α-amino-3-hydroxy-5-methyl-4-isoxazolepropionic acid
AMT =: amitriptyline
APCI =: atmospheric pressure chemical ionization
APPI =: atmospheric pressure photoionization
ARC =: arecoline
AT =: anatabine
BZE =: benzoylecgonine
CAF =: caffeine
CB =: cannabinoid
CBD =: cannabidiol
CBN =: cannabinol
CE =: capillary electrophoresis
CCE =: cocaethylene
CE-MS =: Capillary electrophoresis-mass spectrometry
Ch =: choline
CI =: chemical ionization
CID-MS/MS =: collision-induced dissociation tandem mass spectrometry
cLC-MS =: capillary liquid chromatography–mass spectrometry
CL =: clomipramine
CNO =: cotinine-N-oxide
COC =: cocaine
COD =: codeine
COT =: cotinine
COT-GLU =: cotinine-N-β-d-glucuronide
CSF =: human cerebrospinal fluid
CTP =: citalopram
CZE-ESI-TOF =: capillary zone electrophoresis-electro-
MS =: spray ionization-time of flight mass spectrometry
2D =: two-dimensional
DA =: dopamine
DA-GLU =: dopamine glucuronide
DCL =: desmethylclomipramine
DDMCTP =: didesmethylcitalopram
DDV =: N,O-didesmethylvenlafaxine
DHC =: dihydrocodeine
DHM =: dihydromorphine
DMCL =: desmethylclomipramine
DLX =: duloxetine
DM-CTP =: desmethyl-citalopram
DMD =: desmethyldoxepin
DMDS =: desmethyldosulepin
DMFLU =: desmethylfluoxetine
DMI =: desipramine
DMMIR =: desmethylmirtazapine
DMVEN =: desmethylvenlafaxine
DOPAC =: 3,4-dihydroxyphenylacetic acid
DOS =: dosulepin
DOX =: doxepin
ECNCI =: electron capture negative chemical ionization
EDDP =: 2-ethylidene-1,5-dimethyl-3,3-diphenylpyrrolidine
EI =: electron impact ionization
EME =: ecgonine methylester
ENP =: enalapril
ENPT =: enalaprilat
ESI =: electrospray ionization
ESI-MS =: electrospray ionization mass spectrometry
EP =: ephedrine
FLU =: fluoxetine
FLV =: fluvoxamine
GABA =: gamma-aminobutyric acid
GC =: gas chromatography
GC-FID =: gas chromatography/flame ionization detector
GC/ECNCI/MS =: gas chromatography/electron capture negative chemical ionization mass spectrometry
GC-MS =: gas chromatography mass spectrometry
Glu =: glutamate
GLU =: glucuronide
GLUs =: glucuronides
KT =: ketamine
HFBA =: heptafluorobutyric acid
HF-LPME =: hollow fiber liquid-phase micro-extraction
5-HIAA =: 5-hydroxyindoleacetic acid
HILIC =: hydrophilic interaction chromatography
HPLC =: high performance liquid chromato-graphy
HPLC-ESI =: MS-high performance liquid chromato-graphy-electrospray ionization mass spectrometry
HPLC-MS =: high performance liquid chromato-graphy-mass spectrometry
HS-SPME =: headspace solid-phase microextraction
5-HT =: 5-hydroxytryptamine
5-HT-GLU =: GLU-5-hydroxytryptamine-glucuronide
5-HTP =: 5-hydroxytryptophan
HVA =: homovanillic acid
IC =: Ion-exchange chromatography
IDRA21 =: 7-chloro-3-methyl-3,4-dihydro-2H-1,2,4-benzothiadiazine 1,1-dioxide
IM =: imipramine
IsoACh =: (3-carboxylpropyl)-trimethylammonium
LAE =: lysergic acid ethylamide
LC =: liquid chromatography
LC-MS =: liquid chromatography mass spectro-metry
LC-ESI-MS =: liquid chromatography electrospray ionization mass spectrometry
LEO =: lysergic acid ethyl-2-hydroxyethyl-amide
LLE =: liquid-liquid extraction
LOD =: limit of detection
LOQ =: limit of quantification
LSD =: lysergic acid diethylamide
MA =: methamphetamine
MAM =: 6-methylacetylmorphine
MAT =: maprotiline
MBDB =: N-methyl-1-(1,3-benzodioxol-5-yl)-2-butanamine
MC =: mescaline
MCC =: metacerebral cell
MD =: methadone
MDOAM =: methylene dioxyamphetamine
MDOEAM =: methylene dioxyethyl amphetamine
MDOMAM =: methylene dioxymethyl amphetamine
MeOH =: methanol
MEL =: melatonin
MECA =: mecamylamine
MIA =: mianserin
MIL =: milnacipran
MIR =: mirtazapine
MLT =: melatonin (check in text)
MN =: metanephrine
MP =: morphine
MOC =: moclobemide
MRM =: multiple reaction monitoring
MS =: mass spectrometry
MSPE =: micro-solid phase extraction
3-MT =: 3-methoxytyramine
NC =: norcotinine
NCI =: negative chemical ionization
NCOD =: norcodeine
NDHC =: nordihydrocodeine
NDHM =: nordihydromorphine
NDV =: N-desmethylvenlafaxine
NE =: norepinephrine
NF =: norfluoxetine
NIC =: nicotine
NIC-GLU =: nicotine-N-β-d-glucuronide
NK =: norketamine
NN =: nornicotine
NNNO =: nornicotine N-oxide
NNO =: nicotine-N-oxide
NMP =: normorphine
NMN =: normetanephrine
NOCOD =: noroxycodone
NOMOR =: noroxymorphone
NOR =: nortriptyline
NSAL =: norsalsolinol
OCOD =: oxycodone
ODV =: O-desmethylvenlafaxine
OH-COT =: trans-3-hydroxy cotinine
11-OH-THC =: 11-hydroxy-THC
OMOR =: oxymorphone
PCI =: positive chemical ionization
PCP =: phencyclidine
PSC =: psilocybine
PXT =: paroxetine
SAL =: salsolinol
SDME =: single drop microextraction
SIOMS =: surface ionization organic mass spectrometry
SIR =: selected ion recording
SMPE =: solid-phase microextraction
SPE =: solid-phase extraction
SRM =: selected reaction monitoring
SRT =: sertraline
RBX =: reboxetine
RSD =: relative standard deviation
TCAs =: tricyclic antidepressants
TFA =: trifluoroacetic acid
THC =: Δ9-tetrahydrocannabinol
THCCOOH =: 11-nor-9-carboxy-THC
THC-GLU =: THC-glucuronide
TOF =: time of flight
TOF-MS =: time of flight-mass spectrometry
TP =: l-tryptophan
trans-OH-COT =: trans-3-hydroxycotinine
trans-NICO =: trans-nicotine-1′-oxide
TRZ =: trazodone
UPLC-ESI-MS =: ultra performance liquid chromato-graphy-electrospray ionization mass spectrometry
UHPLC =: ultra high performance liquid chromato-graphy
VCL =: varenicline
VEN =: venlafaxine
